# Development and validation of the Kilifi Stigma Scale for Epilepsy in Kenya

**DOI:** 10.1016/j.yebeh.2012.02.019

**Published:** 2012-05

**Authors:** Caroline K. Mbuba, Amina Abubakar, Peter Odermatt, Charles R. Newton, Julie A. Carter

**Affiliations:** a KEMRI/Wellcome Trust Research Programme, Centre for Geographic Medicine Research (Coast), Kilifi, Kenya; b Tilburg University, The Netherlands; c Utrecht University, The Netherlands; d Swiss Tropical and Public Health Institute, Basel, Switzerland; e University of Basel, Basel, Switzerland; f London School of Hygiene and Tropical Medicine, London, UK; g Neurosciences Unit, Institute of Child Health, University College London, London, UK; h Department of Psychiatry, University of Oxford, Oxford, UK; i Centre for International Health and Development, Institute of Child Health, University College London, London, UK

**Keywords:** Epilepsy, Stigma scale, Tool development, Validation, Kenya

## Abstract

The aim of this study was to develop and validate a tool to measure perceived stigma among people with epilepsy (PWE) in Kilifi, Kenya. We reviewed existing scales that measured stigma, particularly of epilepsy. We conducted a qualitative study to determine salient concerns related to stigma in Kilifi. Themes were generated, and those related to stigma were used to construct an 18-item stigma scale. A descriptive cross-sectional survey was then conducted among 673 PWE to assess the reliability and validity of the scale. Internal consistency was calculated using Cronbach's alpha and test–retest reliability with an interclass correlation coefficient. The final scale had 15 items, which had high internal consistency (Cronbach's *α* = 0.91) and excellent test–retest reliability (r = 0.92). Factor analysis indicated that the scale was unidimensional with one factor solution explaining 45.8% of the variance. The Kilifi Stigma Scale for Epilepsy is a culturally appropriate measure of stigma with strong psychometric properties.

## Highlights

► We developed and validated a scale to measure perceived stigma in epilepsy. ► The scale was unidimensional: one factor solution explained 45.8% of the variance.► It had high internal consistency and excellent test-retest reliability. ► It can be adapted and validated for use in other research settings.

## 1 Introduction

Stigma associated with epilepsy is common in many cultures [1] and is considered to be one of the most important factors having a negative influence on the lives of people with epilepsy (PWE) and their families [2–5]. It erodes individuals' social status, social networks and self-esteem, all of which contribute to poor outcomes such as isolation, unemployment, lower prospects of marriage and not seeking treatment [6,7]. PWE report that dealing with stigma and the associated prejudicial responses from others is one of their largest challenges [8]. Consequently, the Global Campaign Against Epilepsy “Out of the Shadows” project has focused attention on epilepsy-associated stigma [9].

Stigma related to epilepsy fits well into Weiss and Ramakrishna's definition of “a social process or related personal experience characterized by exclusion, rejection, blame, or devaluation that results from experience or reasonable anticipation of an adverse social judgment about a person or group identified with a particular health problem” [10]. Thus, epilepsy-associated stigma can be understood by distinguishing between ‘enacted’ and ‘perceived’ stigma. Enacted stigma refers to discrimination against PWE on the grounds of their social unacceptability, whereas perceived stigma refers to the shame felt by PWE and the fear of anticipated discrimination [11–13].

Stigma is a complex concept to investigate in PWE because it involves personal attitudes and beliefs, elements of secrecy and disclosure management, as well as influences from the social environment [14]. Tools to measure perceived stigma among PWE have been developed mostly in Western countries [7,8,12,14–18] and middle income countries [19–22]. The most commonly used scale in epilepsy is a three-item scale developed by Jacoby [12] though it has been shown to produce disparate results in Western and low income countries [23–27]. This highlights the fact that cultural perceptions and values play an important role in understanding the concept and content of stigma by an individual. Therefore, in designing a reliable and valid tool, it is essential to accommodate the cultural beliefs and to understand the target group.

Taking this into consideration, we developed and validated a tool to measure perceived stigma among PWE in Kilifi, Kenya. Using Cronbach's alpha and an intraclass correlation coefficient, we investigated reliability [28]. Moreover, various forms of validity were evaluated. Factorial structure and correlation with age and sex were used to evaluate construct validity [12,14]. Discriminant validity was investigated by examining the differences in mean scores between those who reported physical and sexual abuse and those who experienced severe symptoms of epilepsy. A study in Zambia showed that sexual abuse increased stigma because women with epilepsy were abandoned by their male partners who would serve as protectors [29]. They were also neglected, physically abused and ejected from family homes [29]. Based on this prior research, it is expected that those who experience abuse and those who experience severe symptoms of epilepsy are more likely to report higher perceived stigma. Three hypotheses were formulated:

1) There would be no differences in stigma scores by sex, but we anticipated significant differences by age;
2) PWE who experienced abuse, whether physical or sexual, would report a much higher level of perceived stigma compared to those who had not experienced abuse;
3) PWE with frequent seizures would report higher level of perceived stigma, but type of epilepsy would have no effect on the stigma scores.

## 2 Methods

### 2.1 Study site and previous work

The development of the scale took place in the Kilifi Health Demographic Surveillance System (KHDSS), where 86% of PWE were not receiving treatment from health facilities [30], which could partially be explained by the cultural beliefs and attitudes held by PWE [31]. A previous study suggested that PWE were more likely to consult traditional healers (THs) due to misconceptions and superstitions associated with epilepsy [32]. However, these studies did not assess the level of stigma experienced by PWE. Therefore, we developed and validated the Kilifi Stigma Scale for Epilepsy (KSSE) that can be used to measure perceived stigma among PWE in Kilifi.

### 2.2 Development of the Kilifi Stigma Scale for Epilepsy

The items for the scale were developed in three phases: (1) formative research and concept development; (2) item development and validity assessment and (3) evaluating the scale in PWE.

#### 2.2.1 Phase 1: formative research and concept development

First, we reviewed the literature to examine the existing scales that measured perceived stigma [7,8,12,14–22]. Second, we conducted a qualitative study with PWE and their caregivers to identify issues related to stigma in Kilifi. Focus group discussions (FGDs) and in-depth interviews were conducted by three trained interviewers fluent in the local language. The interviews were recorded, translated and transcribed. The data were entered onto N-VIVO qualitative analysis software (version 9, QSR; Melbourne, Vic, Australia; http://www.qsrinternational.com/) to enable storage, organization and retrieval. Data were analyzed using framework analysis, as described by Ritchie and Spencer [33]. Themes were independently generated from the data by two researchers (CKM and JAC), and once thematic consensus was reached, all the data were coded. This process served to maximize the rigor and validity of the analysis. The themes related to stigma were then used to investigate in phase 2.

#### 2.2.2 Phase 2: item development and validity assessment

##### 2.2.2.1 Item generation

The project team held discussions about the concepts to include, an appropriate response scale and the overall structure of the questionnaire. An initial first version was developed that contained 18 items that were considered to cover the most important aspects of perceived stigma. Eight of the 18 items in the KSSE were taken directly or revised from questions used in previous studies investigating stigma in epilepsy [7,12,19,20]. The remaining ten items were developed from thematic analysis of FGDs and in-depth interviews. The scale was developed in English and went through a process of translation and back-translation into the local dialect, Kigiriama, to ensure consistency and accuracy.

##### 2.2.2.2 Scoring the scale

A simple three-point Likert scoring system was employed to make it as easy as possible to respond to the items [34]. The PWE were asked to respond to each item by stating how much they thought a particular aspect of their life was affected by epilepsy. Responses were scored as follows: “not at all” (score of 0), “sometimes” (score of 1) and “always” (score of 2). A total score was calculated by addition of all item scores. The higher the score, the greater was the sense of perceived stigma.

##### 2.2.2.3 Face validation of the scale

This was assessed to determine if the questionnaire contained relevant items for assessing perceived stigma in our context. Two clinicians and five research assistants, who were Kigiriama speaking, familiar with the culture and had experience in epilepsy, were asked to evaluate the relevance, clarity and conciseness of the items included in the questionnaire. Seven respondents were of the opinion that the questions measured perceived stigma. Based on this initial assessment, all 18 items were retained.

The questionnaire was then pilot tested with six PWE and seven caregivers of PWE. It was administered by two interviewers fluent in the local language. The respondents were asked to: (a) comment on whether the items measured perceived stigma; (b) rate the items on a three-point rating scale (0 = not at all, 1 = sometimes, 2 = always); (c) provide explanation supporting their decision to assign a rating to an item; and (d) comment on the clarity and flow of the questions. The outcome of the pilot indicated that all 18 items were clear to the respondents and no item was revised. The minimum score on the scale was 0 and maximum was 2, whereas the individual items had means ranging from 0.30 to 1.69.

#### 2.2.3 Phase 3: evaluating the scale

A descriptive cross-sectional survey was conducted in the KHDSS to further assess the reliability and validity of the scale. Six hundred and seventy three PWE completed the scale, of whom 203 were PWE and 470 were caregivers of children with epilepsy (CWE). Of the 673 PWE who completed the scale, 499 (74.1%) reported seeking treatment for epilepsy from a health facility, whereas 174 (25.9%) reported never seeking treatment. All the PWE included in the study had active convulsive epilepsy and were indentified through an epidemiological survey. The test–retest reliability of the scale was evaluated with a subset of 70 PWE: the interviewer administered the scale twice to these respondents at an interval of 3 weeks.

### 2.3 Ethical considerations

Written informed consent was obtained from all study participants. Where the PWE was a child or an adult who could not respond, a caregiver was interviewed. Approval for the study was obtained from the Kenya Medical Research Institute/National Ethical Review Committee.

### 2.4 Data analysis

Data were double entered in MySQL and verified before being transferred to SPSS (version 15, SPSS Inc., Chicago) for analysis. Descriptive statistics were generated to evaluate the score distribution per response category. The internal consistency of the scale was calculated using Cronbach's alpha (*α*) [28]. An interclass correlation coefficient was used to evaluate the test–retest reliability. Factor analysis using varimax rotation was performed to examine the structure of the scale. Items were retained if they had an item-total correlation ≥ 0.2 and a factor loading ≥ 0.40 [35,36]. Correlation analysis was used to evaluate the relationship between the scale scores and sex, age, history of physical and sexual abuse, seizure frequency and types of seizures.

## 3 Results

### 3.1 Study participants

Six hundred and seventy three PWE completed the scale, of whom 51.0% were men. The majority of PWE, 393 (58.1%), were children aged 18 years and below. Among adults, 133 (47.5%) had no formal education, and only 8 (2.9%) had tertiary level of education. The largest faith group was traditional, which is composed of 297 (44.1%), meaning most PWE have traditional religious beliefs (Table 1). Of the 673 PWE, 174 (44.3%) of the children had 1–6 seizures in three months, whereas a similar number of seizures were reported by 140 (50.0%) of the adults.

### 3.2 Psychometric properties

#### 3.2.1 Descriptives

The descriptive statistics show that for most items, participants responded “not at all” to most of the items on the stigma scale, indicating that they did not feel stigmatized (Table 2). However, higher perceived stigma was reported on three items (item 13: not relating well with family members, item 14: not being accepted by peers and item 17: not being taken seriously by other people) (Table 2).

#### 3.2.2 Internal consistency

The alpha score for the whole scale (18 items) was 0.85. This initial analysis demonstrated that three items had a negative total correlation: not relating well with family members (item 13: − 0.02), not being accepted by peers (item 14: − 0.46) and not being taken seriously by other people (item 17: − 0.27). After exclusion of these items, the remaining 15 items had excellent internal consistency (*α* = 0.91). The internal consistency of the scale is outlined in Table 3. Given that we had two different samples, we split the data based on who responded to the questionnaire (203 PWE and 470 caregivers of CWE). There was no difference in internal consistency based on who responded (0.90) for PWE or (0.91) for caregivers of CWE.

#### 3.2.3 Test–retest reliability

Test–retest reliability was estimated by calculating the inter-correlation coefficient and found to be excellent (r = 0.93).

#### 3.2.4 Factor analysis

The dimensionality of the scale was studied using factor analysis. All the items loaded on one factor, which accounted for 45.8% of the variance (eigenvalue = 6.87). The consistently high factor loadings (0.46–0.82) strongly supported one unitary construct of the scale, as shown in Table 4. These results support the use of a summated score to represent an overall index called ‘perceived stigma’.

#### 3.2.5 Construct validity

Correlations were calculated to explore the relationship between socio-demographic characteristics and stigma scores. Results indicated that there was no relationship between sex and perceived stigma scores (r = 0.04, p = 0.30). High correlations were found between age and perceived stigma scores, with younger age associated with greater perception of stigma (r = 0.68, p = 0.03). Our analysis confirmed that there was a moderate correlation between perceived stigma scores and physical abuse (r = 0.33, p < 0.001) as well as perceived stigma scores and sexual abuse (r = 0.76, p < 0.01). There was also a moderate correlation between perceived stigma and seizure frequency (r = 0.58, p < 0.01) but not between perceived stigma and type of seizures (r = 0.02, p = 0.23).

## 4 Discussion

The purpose of this study was to develop and evaluate a culturally appropriate measure of perceived stigma among PWE in a rural Kenyan setting. Using a systematic approach to tool development, as previously used in Kilifi [37–39], we developed a 15-item scale that provides a measure of stigma in epilepsy with proven reliability and validity.

### 4.1 Reliability

Criteria described by Cicchetti were employed in evaluating the level of acceptability of the observed values of reliability coefficients [40]. A correlation of 0.70 or higher is usually considered an acceptable level of internal consistency [7,40].

The excellent internal consistency and retest reliability observed in this study supported the utility and reliability of the tool in our setting. Furthermore, the selection of culturally appropriate items through qualitative research ensured that the items were appropriate to this context. The reliability of the KSSE compares well to other scales developed to measure stigma in epilepsy [7,14,19,20].

### 4.2 Validity

Factor-analytic evidence suggests that the scale is unidimensional, indicating that it measured only one construct. Internal consistency did not differ whether it was a PWE who responded or a caregiver of CWE. This suggests that perceived stigma of children or PWE with neuro-cognitive impairment can be assessed through a caregiver (mother, father or guardian) using the same scale.

Analysis was also conducted to examine the correlation between the demographic variables and stigma scores. Results indicated that sex was not correlated with the stigma scores. However, we observed an association between perceived stigma and age, which provides support for the age sensitivity of the scale.

Younger age was associated with greater perception of stigma, a finding also reported in other studies [12,14,16]. Possibly, older people were less likely to report feeling stigmatized because discriminatory attitudes toward epilepsy may have less significance to them than younger people, who want to fit in with peers. Given its potentially negative impact, there is a need to address stigma as part of a comprehensive care system, especially for young PWE.

Further analysis was conducted to correlate perceived stigma and reported abuse. Consistent with our hypothesis, PWE who experienced physical or sexual abuse were more likely to report perceived stigma. This provides further evidence of the discriminant validity of the tool. Moreover, it highlights other aspects of the psychosocial needs of PWE. A study from Zambia reported that PWE experienced higher rates of physical abuse and that women with epilepsy are more likely to have experienced sexual abuse [27,29]. There is a need for further studies to examine the prevalence and impact of abuse of PWE. However, such studies should also investigate physical and sexual abuse in a detailed manner in order to accurately assess the level of abuse. Our study may have underestimated the problem since we asked a single (yes/no) question regarding physical and sexual abuse and since it is a sensitive issue people may be reluctant to disclose it.

An additional approach to validation was to correlate perceived stigma and seizure variables. As hypothesized, PWE who had more seizures experienced higher perceived stigma than those who had fewer seizures. This could mean that severity of epilepsy has an effect on the level of stigma perceived by PWE in Kilifi. Our results are consistent with other studies that have shown that greater perceptions of stigma were associated with greater seizure severity [14,17,23]. There was no correlation between seizure type and stigma scores, suggesting no difference in perceived stigma between PWE who had generalized seizures and those with focal seizures. This contrasts with a study by Austin et al. which found a difference in stigma scores between parents of children with absence and partial generalized seizures. However, this difference was observed only among children who had epilepsy for a long time (chronic sample) and not in children who had new-onset epilepsy.

## 5 Limitations

The ‘gold standard’ measure of assessment validity is concurrent validity, but in common with most assessment development in African settings where there are few existing validated measures, there was no comparable assessment available for this analysis.

## 6 Conclusion

The KSSE is a culturally appropriate measure with strong psychometric properties and could be adapted and validated for use in other settings. It can be administered to PWE or their caregivers. The scale will help researchers assess perception of stigma in epilepsy and measure how this changes over time. The scale also allows objective quantification, which can be used to assess public health interventions aimed at reducing stigma.

## Figures and Tables

**Table 1:** Demographic characteristics of study participants.

Variable	Children n = 393	Adults n = 280
Age years: n (%)		
1–5	92 (23.4)	n/a
6–10	111 (28.2)	n/a
11–18	190 (48.4)	n/a
19–30	n/a	155 (55.4)
> 30	n/a	125 (44.6)
Sex: n (%)		
Female	184 (46.8)	148 (52.9)
Male	209 (53.2)	132 (47.1)
Religion: n (%)		
Christian	167 (42.5)	128 (45.7)
Islam	52 (13.2)	29 (10.4)
Traditional	174 (44.3)	123 (43.9)
Educational level: n (%)		
None	173 (44.0)	133 (47.5)
Primary	194 (49.4)	122 (43.5)
Secondary	26 (6.6)	17 (6.1)
Tertiary	n/a	8 (2.9)
Occupation: n (%)		
Farmer	n/a	150 (53.6)
Trader	n/a	46 (16.4)
Casual	n/a	34 (12.1)
Other	n/a	50 (17.9)
Marital status: n (%)		
Single	n/a	77 (27.5)
Married	n/a	142 (50.7)
Separated	n/a	7 (2.5)
Divorced	n/a	17 (6.1)
Widowed	n/a	37 (13.2)

n/a: not applicable.

**Table 2:** Proportion of responses by study participants (n = 673).

Item	Not at all (%)	Sometimes (%)	Always (%)
1. Do you feel different from other people?	36.1	37.4	26.5
2. Do you feel lonely?	59.8	27.9	12.3
3. Do you feel embarrassed?	65.3	23.3	11.4
4. Do you feel disappointed in yourself?	42.0	39.7	18.3
5. Do you feel you cannot have a rewarding life?	62.0	22.7	15.3
6. Do you feel you cannot contribute anything in society?	60.9	21.6	17.5
7. Do you feel you cannot join others in public places?	56.9	19.8	23.3
8. Do you feel other people are uncomfortable with you?	59.7	29.1	11.2
9. Do you feel other people don't want to go to occasions with you?	57.4	27.6	15.0
10. Do you feel other people treat you like an inferior person?	54.7	26.7	18.6
11. Do you feel other people would prefer to avoid you?	62.2	25.6	12.2
12. Do you feel other people avoid exchanging greetings with you?	75.6	18.1	6.3
13. Do you feel you do not relate well with your family?	21.4	11.9	66.7
14. Do you feel you are not accepted by your peers?	6.7	18.0	75.3
15. Do you feel you are mistreated by other people?	67.8	24.8	7.4
16. Do you feel other people discriminate against you?	67.3	23.3	9.4
17. Do you feel other people do not take you seriously?	8.8	18.9	72.3
18. Do you feel other people treat you like an outcast?	69.4	19.9	10.7

**Table 3:** Internal consistency of the Kilifi Stigma Scale for Epilepsy (n = 673).

Item	Scale mean if item deleted	Scale variance if item deleted	Corrected item-total correlation	Alpha if item deleted
1. Different	7.27	44.6	0.53	0.91
2. Lonely	7.65	44.8	0.59	0.90
3. Embarrassed	7.72	45.7	0.49	0.91
4. Disappointed	7.41	45.8	0.44	0.91
5. Rewarding life	7.64	45.9	0.42	0.91
6. Society	7.61	44.7	0.53	0.91
7. Public places	7.51	44.2	0.53	0.90
8. Uncomfortable	7.66	43.5	0.75	0.89
9. Occasions	7.60	42.9	0.74	0.89
10. Inferior	7.54	42.5	0.75	0.89
11. Avoid	7.68	43.3	0.75	0.89
12. Greetings	7.87	45.2	0.67	0.90
13. Mistreated	7.78	45.4	0.59	0.90
14. Discriminate	7.76	44.2	0.70	0.90
15. Outcast	7.76	44.5	0.64	0.90

Items were preceded with the following phrase “Do you feel…” as indicated in Table 2.

**Table 4:** Factor loading of the fifteen items of the Kilifi Stigma Scale for Epilepsy (n = 673).

Item	Factor loading
1. Different	0.58
2. Lonely	0.63
3. Embarrassed	0.54
4. Disappointed	0.48
5. Rewarding life	0.46
6. Society	0.57
7. Public places	0.58
8. Uncomfortable	0.81
9. Occasions	0.81
10. Inferior	0.82
11. Avoid	0.82
12. Greetings	0.74
13. Mistreated	0.67
14. Discriminate	0.77
15. Outcast	0.72

Items were preceded with the following phrase “Do you feel…” as indicated in Table 2.
